# *Trichoderma harzianum* inoculation promotes sweet sorghum growth in the saline soil by modulating rhizosphere available nutrients and bacterial community

**DOI:** 10.3389/fpls.2023.1258131

**Published:** 2023-09-12

**Authors:** Yanli Wei, Han Yang, Jindong Hu, Hongmei Li, Zhongjuan Zhao, Yuanzheng Wu, Jishun Li, Yi Zhou, Kai Yang, Hetong Yang

**Affiliations:** ^1^ Ecology Institute of Qilu University of Technology (Shandong Academy of Sciences), Jinan, China; ^2^ School of Agriculture, Food and Wine, The University of Adelaide, Urrbrae, SA, Australia

**Keywords:** *Trichoderma harzianum*, sweet sorghum, available nutrients, bacterial community, N-cycling genes

## Abstract

As one of the major abiotic stresses, salinity can affect crop growth and plant productivity worldwide. The inoculation of rhizosphere or endophytic microorganisms can enhance plant tolerance to salt stresses, but the potential mechanism is not clear. In this study, *Trichoderma harzianum* ST02 was applied on sweet sorghum [*Sorghum bicolor* (L.) Moench] in a field trial to investigate the effects on microbiome community and physiochemical properties in the rhizosphere soil. Compared with the non-inoculated control, *Trichoderma* inoculation significantly increased the stem yield, plant height, stem diameter, and total sugar content in stem by 35.52%, 32.68%, 32.09%, and 36.82%, respectively. In addition, *Trichoderma* inoculation improved the nutrient availability (e.g., N, P, and K) and organic matter in the rhizosphere soil and changed the bacterial community structure and function in both bulk and rhizosphere soil by particularly increasing the relative abundance of Actinobacter and N-cycling genes (*nifH*, archaeal and bacterial *amoA*). We proposed that *T. harzianum* ST02 could promote sweet sorghum growth under saline conditions by regulating available nutrients and the bacterial community in the rhizosphere soil.

## 1 Introduction

The issue of soil salinization is a pressing environmental concern, with an estimated 20% of irrigated land worldwide affected by salinity (Yuan et al., 2016). Although plants have developed various strategies to withstand salt stress (Rojas-Tapias et al., 2012), these mechanisms are insufficient to support growth under severe salt stress conditions. Moreover, the intricate interplay between many microorganisms and plants (Hassani et al., 2018) has a positive impact on the viability and overall health of plants (Rosier et al., 2016; Etesami and Maheshwari, 2018). It has been found that the rhizosphere microorganisms can interact with their hosts to form a root–soil–microbial interaction network, which can detect and react to signal molecules released by plant roots, leading to enhanced tolerance to biotic and abiotic stress factors such as salinity (Qin et al., 2016; Zhang et al., 2017a). Myriad rhizosphere or endophytic microorganisms can effectively improve the salt tolerance of plants by modulating the ion balance (Zhu et al., 2016) and/or increasing osmotic protective substances in plants (Bhattacharyya et al., 2015). Microorganisms can also produce 1-aminocyclopropane-1-carboxylate (ACC) deaminase, siderophores, or indole acetic acid (IAA) to trigger plant defense and metabolic pathways to tolerate salt stress (Xiong et al., 2019).

*Trichoderma* spp. have been extensively studied as antagonistic fungal agents against plant pathogens as well as plant growth promoters (Woo et al., 2014). In addition, there have been numerous reports of applying *Trichoderma* spp. to exert beneficial effects on enhancing plant tolerance under saline conditions (Contreras-Cornejo et al., 2016). Some *Trichoderma* strains can stably colonize plant roots, cause substantial changes in plant metabolism by producing secondary metabolites, and induce plant resistance to abiotic stress (Mastouri et al., 2010). For instance, *T. virens* (Tv29.8) and *T. atroviride* (IMI 206040) enhanced root development of *Arabidopsis thaliana* through auxin signaling and produced osmotic substances to promote plant growth in saline conditions (Contreras-Cornejo et al., 2014). *T. echinospora* Q1 alleviated the salt stress on cucumber by changing plant hormone levels and phosphate solubilization capacity (Zhao and Zhang, 2015). *T. longibrachiatum* TL-6 disturbed the intracellular ionic homeostasis and modulated the transcriptional levels of IAA and ethylene synthesis genes in wheat seedlings under salt stress through promoted ACC deaminase activity and increased IAA production (Zhang et al., 2019). *Trichoderma* spp. were capable of inhibiting the growth of plant pathogens. For example, the antagonistic activity test of a *T. cyanodichotomus* strain showed that its inhibition ability was high to suppress the growth of *Botryosphaeria dothidea*, *Pythium aphanidermatum*, *Rhizoctonia solani*, and *Verticillium dahlia*, but poor for *Botrytis cinerea* and *Helminthosporium sativum* (Li et al., 2018). Therefore, antagonistic activity of *Trichoderma* may change the community structure, consequently altering the microbial diversity of soil (Pang et al., 2017; Velmourougane et al., 2017). However, the mechanism of *Trichoderma* spp. modulating rhizosphere available nutrients and the microbiome community under saline conditions remains poorly understood.

Sweet sorghum [*Sorghum bicolor* (L.) Moench] is a widely adapted sugar crop with high biomass production and high carbohydrate content (Guo et al., 2018). The sugar-rich stalk of sweet sorghum makes the crop particularly amenable to direct fermentable sugar extraction, and appropriate saline stress can increase sugar accumulation (Sui et al., 2015). The low input costs of sweet sorghum are based on its highly drought tolerance and C4 photosynthesis (Calviño and Messing, 2012). Previously, we isolated *T. harzianum* strain ST02 from the saline soil of the Yellow River Delta in Dongying City and demonstrated that ST02 was able to enhance the tolerance of tomato to NaCl stress in a greenhouse experiment through improving the antioxidant defense reaction of plants (Zhao et al., 2021). Hence, in the present study, we aimed to investigate the impact of ST02 inoculation on physiological growth, biomass, and sugar content of sweet sorghum, along with the changes of nutrient availability and microbiome community in the rhizosphere soil.

## 2 Materials and methods

### 2.1 Plant and microorganism

The seeds of sweet sorghum, variety Aertuo326, were purchased from Hunan Longping Seeds Industry Co. (Changsha, China). *Trichoderma harzianum* ST02 was isolated from saline soil collected at the Yellow River Delta in Dongying City, as described previously (Zhao et al., 2021). Conidial suspension of ST02 was scraped from a potato dextrose agar (PDA) plate cultured for 72 h, and the concentration of conidia was adjusted to 10^8^ CFU mL^−1^ with sterile water. The inoculum for field experiment was prepared as wettable powders composed of 10% fresh conidial suspension (2 × 10^8^ CFU g^−1^) and 90% sterilized diatomite.

### 2.2 Field experiment

The site of field experiment was located in Guangrao town, Dongying City, Shandong Province (118°37′5ʺE, 37°15′18ʺN). The soil was classified as sandy loam, and the physicochemical characteristics were pH (soil/water ratio of 1:2.5, w/v) of 7.15, electric conductivity of 585 μS cm^−1^, organic matter of 3.98 mg g^−1^, total N of 1.39 mg g^−1^, total P of 0.46 mg g^−1^, and total K of 11.81 mg g^−1^. The field experiment was conducted in a completely randomized block design with +/− *Trichoderma* inoculation as two factors. For the + *Trichoderma* treatment, 3 kg of ST02 wettable powder was mixed with 20 kg of sweet sorghum seeds before sowing. For the − *Trichoderma* control, the same amount of sterilized water was mixed with sterilized diatomite to prepare the wettable powder for the application. The field was divided into eight plots (5 m × 30 m) by 0.5-m gaps; four plots were chosen at random as + *Trichoderma* treatment and the other four plots as − *Trichoderma* control. Each treatment was sown with 12 rows by a seeding machine. Conventional agricultural management measures were adopted after sowing in all plots.

To determine the inoculum amount of *T. harzianum* ST02 on each sweet sorghum seed, 50 inoculated seeds were rinsed with 20 mL of 0.9% sterilized sodium chloride solution in a 50-mL triangular bottle (Bazhanov et al., 2017). After being vortexed for 5 min, the resulting suspensions and their serial dilutions were smeared on PDA plates. *Trichoderma* colonies were counted after 3 days at 26°C.

After sweet sorghum maturity and harvesting, 20 plants were randomly selected from each treatment to measure the plant height, stalk diameter, stalk fresh weight, and total sugar content in the stem. Whole sorghum stems were crushed in a cane-juice squeezer, and the collected juice was used to determine the total sugar content by a handheld refractometer (Lichen LC-DR-328, LC-BX Inc., Shanghai, China), which has been widely used to measure sugar content in the liquid solution. Sugar content was indicated by the light reflectance when it passed through the sample and calibrated by the samples with known sugar concentration. The total yield of sweet sorghum at different plots was recorded at the time of harvesting.

### 2.3 Soil sampling

Bulk soil and rhizosphere soil samples were collected at five different sites as a biological replicate, with a total of seven replicates for each treatment (28 samples in total). Bulk soil was designated as the soil of 20 to 30 mm away from the roots, and rhizosphere soil was designated as within 1 mm on the root surface. All soil samples were divided into two parts: one part of 2 g was stored at −80°C for DNA extraction, and the other part of 20 g was air dried and stored at room temperature for physicochemical properties analysis.

### 2.4 Soil physicochemical property analysis

The air-dried soil samples were passed through a 2-mm sieve before the physicochemical property analysis. Soil pH and electric conductivity were tested with a pH meter (Mettler Toledo, Germany) in a 1:2.5 (soil/water ratio of 1:2.5, w/v soil: deionized water W/V) suspension (Qiu et al., 2022). Soil total nitrogen (TN) content was determined using the modified Kjeldahl method (HJ717-2014). The total potassium (TK) content was quantitatively determined with an ultraviolet spectrophotometer (GB/T9836-1988). Soil organic matter (SOM) was measured by redox titration with K_2_Cr_2_O_7_ (HJ615-2011). The alkaline hydrolysis diffusion method was used to determine the available nitrogen (AN) content in the soil (Fu et al., 2019). The available phosphorus (AP) concentration was measured by the molybdenum blue method (Olsen and Sommers, 1982), and the available potassium (AK) was measured by flame photometry in 1 M ammonium acetate soil extracts (NY/T 889-2004).

### 2.5 Soil DNA extraction and Miseq sequencing

PowerSoil DNA Isolation Kit (Qiagen, Germany) was used to extract total genomic DNA from 0.25-g soil samples following the manufacturer’s instructions. The extracted DNA was electrophoresed with 1% agarose gel, and the concentration and purification of the genomic DNA were measured by a NanoDrop 2000 spectrophotometer (Thermo Scientific, United States). The specific primers 341F (5′-CCTACGGGNGGCWGCAG-3′) and 805R (5′-GACTACHVGGGTATCTAATCC-3′) (Klindworth et al., 2012) of the bacterial 16S rRNA gene were used for amplification. The purified products were quantified by a Qubit 3.0 DNA detection kit, and then samples were mixed and homogenized in equimolar ratio and sequenced on an Illumina MiSeq PE300 platform at Shanghai Sangon Bioengineering Co. The bioinformatic analysis of the DNA sequence is referred to Li et al. (2022). Alpha diversity indices, such as Shannon diversity, Chao1 richness, and Faith’s phylogenetic diversity, were calculated to estimate the bacterial diversity within an individual sample.

### 2.6 Quantitative PCR analysis of N-cycling genes

The abundances of five representative N-cycling genes (*nifH*, bacterial *amoA*, archaeal *amoA*, *nirS*, and *nosZ*) were determined by quantitative polymerase chain reaction (qPCR) with the CFX96 (Bio-Rad, Hercules, CA, USA) for the rhizosphere and bulk soil DNA samples. The primer sequences are shown in **Table 1**. The 25-µL qPCR reactions contained 2× Power SYBR^®^ Green PCR Master Mix (TaKaRa, Dalian, China) 12.5 µL, each 10 µM forward and reverse primers 0.5 µL, soil DNA samples 1.0 µL, and sterile DNA-free water 10.5 µL. A standard thermal profile was used: 3 min at 95°C for pre-denaturation, followed by 40 cycles of 10 s at 95°C for denaturation and 30 s at optimal temperature for annealing, then extension at 72°C for 20 s. The copy number of target DNA was calculated according to the standard curves, which were generated from 10-fold serial dilution of a plasmid containing the targeted DNA fragments. Melting curve analysis was conducted after the amplification cycles by setting up the temperature at 95°C, 1 min; 65°C, 1 min; from 65°C for every 0.5°C for 10 s; and then continuous increase to 95°C. Three technical replicates for each sample were used to detect the possible error.

**Table 1:** Sequences of oligonucleotide primers required for quantitative PCR.

Target	Primers	Primer sequence (5′–3′)	Length (bp)	N-cycling functions
Bacterial *amoA*	amoA-1F amoA-1R	GGGGTTTCTACTGGTGGT CCCCTCKGSAAAGCCTTCTTC	494	Bacterial ammonia oxidation (Tourna et al., 2008)
Archaeal *amoA*	CrenamoA23f CrenamoA616r	ATGGTCTGGCTWAGACG GCCATCCATCTGTATGTCCA	593	Archaeal ammonia oxidation (Tourna et al., 2008)
*nifH*	PolF PolR	TGCGAYCCSAARGCBGACTC ATS GCC ATC ATY TCR CCG GA	363	Nitrogen-fixation (Poly et al., 2001)
*nirS*	nirSCd3aF nirSR3cd	AACGYSAAGGARACSGG GASTTCGGRTGSGTCTTSAYGAA	407	Nitrite reductase (Kandeler et al., 2006)
*nosZ*	nosZ1F nosZ1R	WCSYТGTTCМТСGАСАGССАG ATGTCGATCARCTGVKCRTTYTC	243	Nitrous oxide reductase (Kandeler et al., 2006)

### 2.7 Statistical analysis

All the results were expressed as the mean ± standard deviation. Duncan’s multiple range test was used to compare the significant difference at the 0.05 level using SPSS 25.0 (IBM Corporation, Armonk, NY, USA). Correlation analyses were carried out using the Spearman correlation method. Bray–Curtis distance was used to investigate the bacterial communities structural variation and then visualized via principal coordinate analysis (PCoA). Redundancy analysis (RDA) was performed to reveal the association of bacterial communities in relation to environmental factors based on relative abundances of bacterial species at different taxa levels using the “vegan” package in R (Lozupone et al., 2010).

## 3 Results

### 3.1 Sweet sorghum growth influenced by +/− *T. harzianum* ST02 in saline soil field test

**Figure 1:**
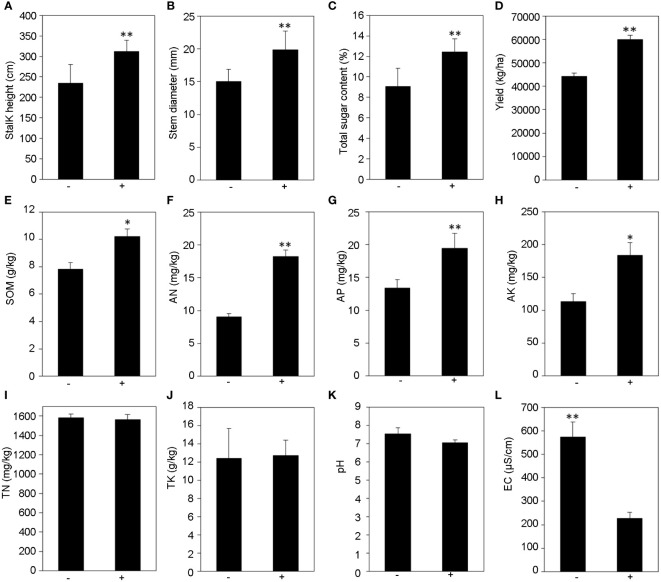
The effect of *T. harzianum* ST02 inoculation on the growth **(A–D)** and rhizosphere soil physicochemical properties **(E–L)** of sweet sorghum. SOM, soil organic matter; TN, total nitrogen; AN, available nitrogen; AP, available phosphorus; TK, total potassium; AK, available potassium; EC, electrical conductivity. The statistically significant values are presented as asterisks (*p < 0.05; **p < 0.01) based on one-way ANOVA with Duncan’s multiple range test. +/− stands for with and without *T. harzianum* ST02 inoculation, respectively.

Field experiments were conducted to study the effects of *T. harzianum* ST02 on the growth of sweet sorghum. The results showed that the application of *Trichoderma* significantly promoted the growth and sugar accumulation of sweet sorghum. It significantly increased in plant height (**Figure 1A**, p < 0.01), stalk diameter (**Figure 1B**, p < 0.01), and sugar content (**Figure 1C**, p < 0.01) by 32.68%, 32.09% and 36.82%, respectively, compared with non-inoculated control. The yield of sweet sorghum stems inoculated with *Trichoderma* ST02 significantly increased by 35.52% to control (**Figure 1D**, p < 0.01).

### 3.2 Analysis of soil physical and chemical properties

The physical and chemical properties of rhizosphere soil in the field experiment were analyzed. Our results indicated that sweet sorghum inoculation with *Trichoderma* had greater contents of SOM, AN, AP, and AK but lower electric conductivity (**Figure 1**) in the rhizosphere soil than the non-inoculated control. Compared with the untreated control, the contents of SOM, AN, AP, and AK in rhizosphere soil increased by 30.6%, 101.55%, 44.77%, and 61.95%, respectively (**Figures 1E–H**). Additionally, the electric conductivity was decreased from 574 μS/cm (− ST02) to 226 μS/cm (+ST02) (**Figure 1L**). For the contents of TN, TK, and pH, there was no significant difference between the soils treated with and without *Trichoderma* (**Figures 1I–K**).

### 3.3 The effects of ST02 inoculation on the soil bacterial community

**Figure 2:**
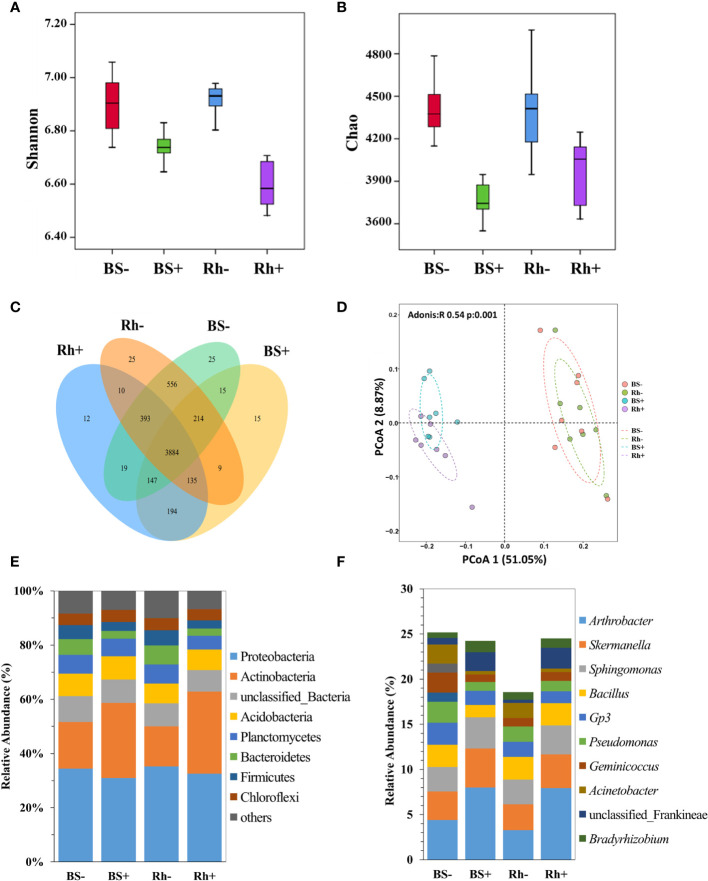
The effect of *T. harzianum* ST02 inoculation on the bacterial community in bulk soil and rhizosphere soil. **(A)** Comparison of Shannon index between groups. **(B)** Comparison of Chao index between groups. **(C)** Venn diagram showing the shared bacterial OTUs. **(D)** PCoA results based on Bray–Curtis distance metric; ellipses are 95% confidence. The relative abundance of bacteria at the **(E)** phylum level and **(F)** genus level. BS−, bulk soil without ST02 inoculation. BS+, bulk soil with ST02 inoculation. Rh−, rhizosphere soil without ST02 inoculation. Rh+, rhizosphere soil with ST02 inoculation. Different letters in **(A, B)** indicate significant differences among the treatments at the p < 0.05 level based on one-way ANOVA with Duncan’s multiple range test.

There were 1,728,736 clean sequences obtained from all samples after filtering with an average length of 416 bp. Shannon indexes and Chao1 indexes of *Trichoderma*-inoculated treatment were significantly lower (**Figures 2A, B**, p < 0.05) than the non-inoculated control in the rhizosphere and bulk soil.

There were 25, 15, 25, and 12 unique OTUs observed in the bulk soil without *Trichoderma* inoculation (BS−), bulk soil with inoculation (BS+), rhizosphere soil without inoculation (Rh−), and rhizosphere soil with inoculation (Rh+) groups (**Figure 2C**), respectively. The PCoA based on the Bray–Curtis distance matrix showed a clear separation of treatments from inoculated with and without *Trichoderma* (**Figure 2D**). The first and second principal coordinates explained the variation of soil bacterial communities to 51.05% and 8.87%, respectively. The bacterial communities of bulk and rhizosphere soils were highly similar and clustered within the inoculation treatment.

According to the taxonomic annotation of OTUs, 12 dominant bacterial phyla (relative abundance >1.0%) accounted for 98.28%–99.12% of the total sequences in the four niches (**Figure 2E**). Proteobacteria, Actinobacteria, Bacteria, Acidobacteria, Planctomycetes, Bacteroidetes, and Firmicutes were the most predominant bacterial phyla with a mean relative abundance >5.0%. *Trichoderma* treatment significantly increased the relative abundances of Actinobacteria, Gemmatimonadota, Nitrospirae, and Acidobacteriota but decreased the relative abundances of Bacteroidetes, Proteobacteria, and Firmicutes in the rhizosphere and bulk soil (**Figure 2E**).

At the genus level, *Arthrobacter* had the highest relative abundance in *Trichoderma*-treated rhizosphere and bulk soil at 7.94% and 7.98%, respectively (**Figure 2F**). In rhizosphere soil, *Trichoderma* inoculation significantly increased the relative abundance of *Arthrobacter*, *Nocardioides*, unclassified Frankineae, and *Bradyrhizobium* by 142.07%, 152.61%, 550.26%, and 23.55%, respectively, but decreased the relative abundance of *Planctomyces*, *Bacillus*, and *Gp3*. In bulk soil, *Trichoderma* inoculation significantly increased the relative abundance of *Arthrobacter*, *Nocardioides*, unclassified Frankineae, and *Bradyrhizobium* by 81.36%, 126.83%, 184.52%, and 107.39%, respectively, but decreased the relative abundance of *Geminicoccus* and *Bacillus*.

### 3.4 Effects of *Trichoderma* inoculation on the abundance of N-cycling genes

**Figure 3:**
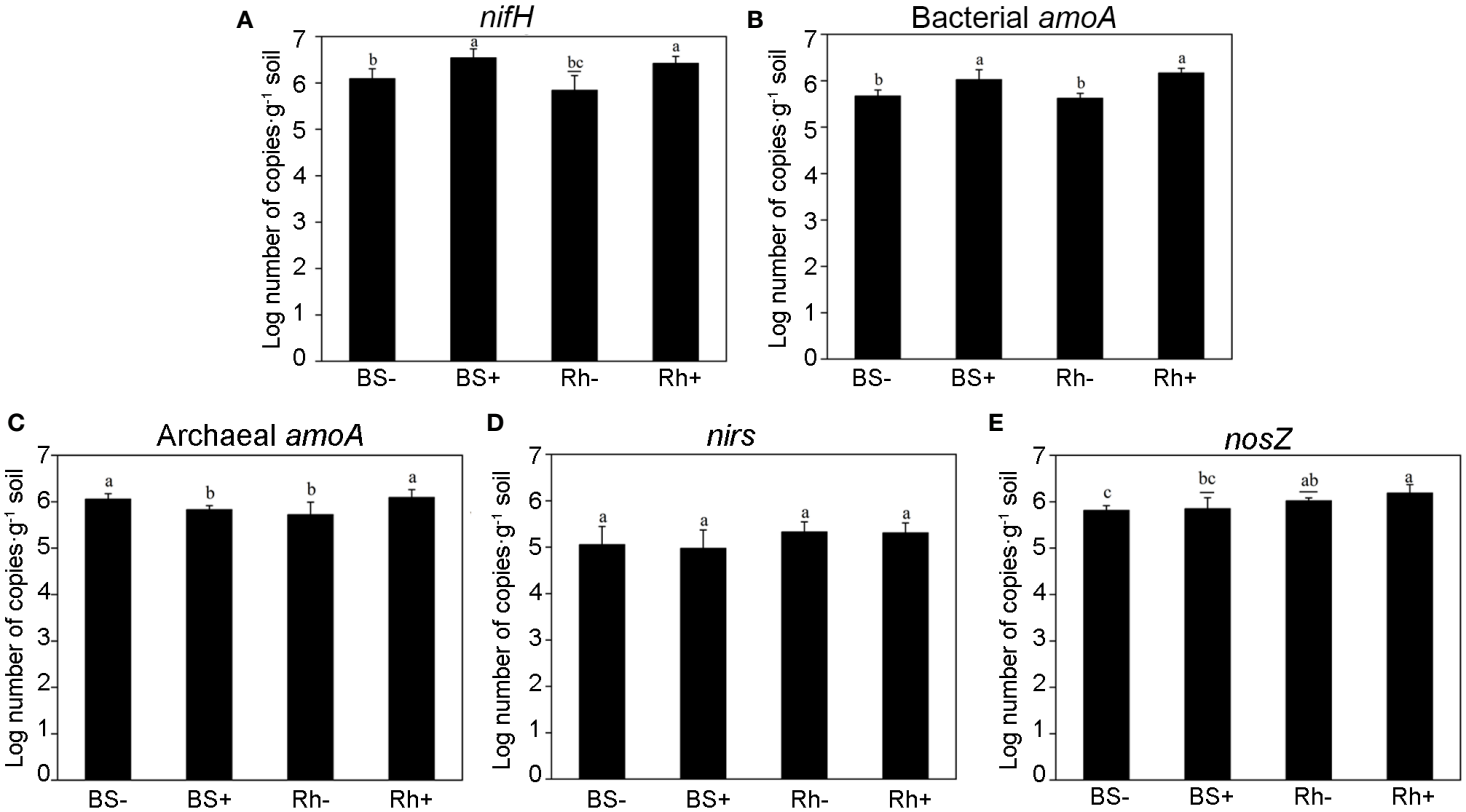
The abundances of nitrogen-cycling genes measured by quantitative PCR. The abundance of five N-cycling genes involved in nitrogen fixation (**A**, *nifH*), ammonia oxidation by bacteria (**B**, bacterial *amoA*) and archaea (**C**, archaeal *amoA*), and denitrification (**D, E**, *nirS*, *nosZ*), in different treatment groups. BS−, bulk soil without inoculation. BS+, bulk soil with inoculation. Rh−, rhizosphere soil without inoculation. Rh+, rhizosphere soil with inoculation. Different letters indicate significant differences among the treatments at the p < 0.05 level based on one-way ANOVA with Duncan’s multiple range test.

The copy numbers of *nifH*, *amoA*, *nirS*, and *nosZ* genes were quantified by qPCR, which related to nitrogen fixation, nitrification, and denitrification, respectively. The results are shown in **Figure 3**. The abundance of *nifH* gene was significantly increased (**Figure 3A**, p < 0.05) in rhizosphere (from 8.88 × 10^5^ to 2.80 × 10^6^ copies) and bulk soil (from 1.35 × 10^6^ to 3.76 × 10^6^ copies) after inoculation with *Trichoderma*, which was 2.15 and 1.78 times greater than control treatments, respectively. The effects of *Trichoderma* inoculation on bacterial *amoA* gene abundance were similar to those of *nifH*, and the copy number of bacterial *amoA* increased significantly in both rhizosphere soil and bulk soil (**Figure 3B**), whereas the archaeal *amoA* was higher in inoculation treatment than the control only in the rhizosphere soil but not the bulk soil (**Figure 3C**). There was no rhizosphere effect or inoculation effect on the abundance of *nirS* (**Figure 3D**). The abundance of *nosZ* gene was greater in the rhizosphere soil than in bulk soil but not influenced by the inoculation effect (**Figure 3E**).

### 3.5 The relationship between rhizosphere soil properties and the structure of rhizobacterial communities

**Figure 4:**
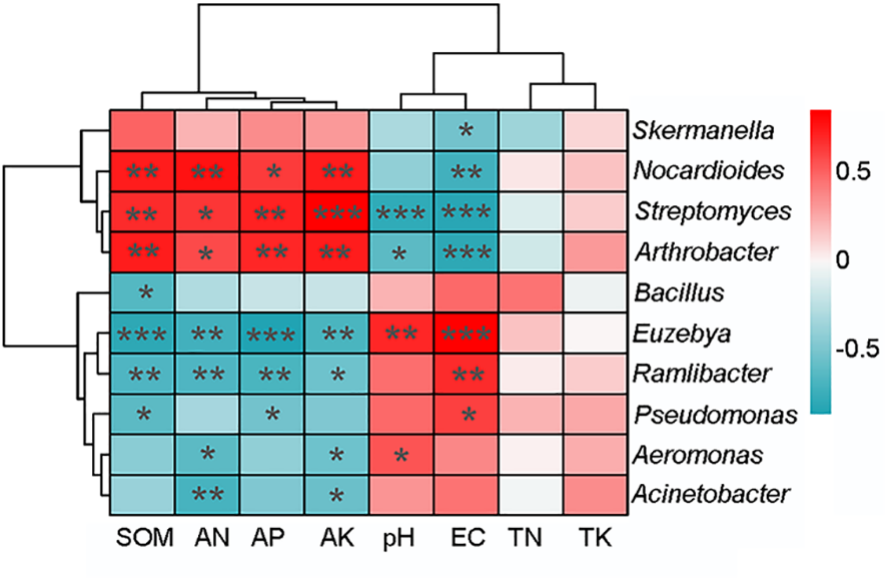
Correlation heatmap between the top 10 bacterial genera and soil properties. The result was based on Spearman’s correlation analysis. Positive relationships are represented in blue, whereas negative relationships are represented in red. The significant correlations are presented as asterisks (*p < 0.05; ** p < 0.01; *** p < 0.001).

Spearman’s correlation analysis was used to test the rhizosphere soil factors influencing the microbial structure and functional pathway. The top 10 dominant bacterial genera were significantly correlated with at least one rhizosphere soil properties (**Figure 4**). The abundances of *Arthrobacter*, *Streptomyces*, and *Nocardioides* were positively correlated with content of SOM, AN, AP, and AK and negatively correlated with EC (p < 0.01), whereas those of *Euzebya* and *Ramlibacter* were negatively correlated with contents of SOM, AN, AP, and AK (p < 0.01) and positively correlated with EC (p < 0.01).

**Figure 5:**
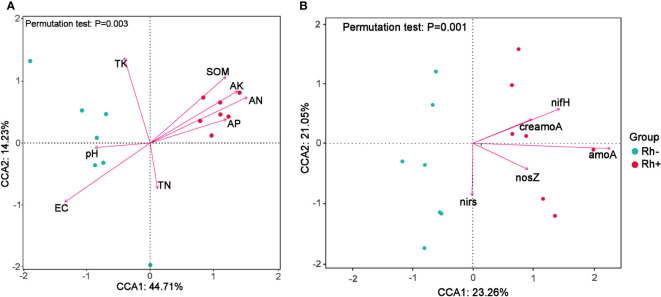
Redundancy analysis (RDA) to test the soil properties **(A)** and the abundance of N-cycling genes **(B)** influencing the bacterial OTU composition in the rhizosphere of sweet sorghum. Rh−, rhizosphere soil without ST02 inoculation. Rh+, rhizosphere soil with ST02 inoculation.

The relationships between bacterial OTU composition and the soil properties were tested by redundancy analysis (**Figure 5A**). The first two axes explained 58.94% of the total variance. The soil SOM, AN, AK, and AP were positively corrected with the rhizosphere inoculated with *Trichoderma*, whereas pH and EC were correlated with non-inoculated control. For the relationships between the abundance of N-cycling genes and bacterial OTU composition (**Figure 5B**), the first two components accounted for 44.31% of the total variance and the inoculation of *Trichoderma* was positively corrected with the abundance of *nifH* and *amoA* in the rhizosphere soil.

## 4 Discussion

Inoculation of beneficial microbes on agricultural crops is a cost-effective way to improve productivity. In this study, we revealed the beneficial effects and possible mechanism of *T. harzianum* ST02 on the growth of sweet sorghum under saline soil. We confirmed that inoculation with *T. harzianum* ST02 improved the shoot length, shoot dry weight, biomass, and total sugar content of sweet sorghum by modifying the rhizosphere soil, including increasing the available nutrients and changing the microbiome structure and function.

### 4.1 *Trichoderma* inoculation increased the growth and yield of sweet sorghum under saline conditions

Promotion of plant growth by *Trichoderma* under salt stress in wheat (Fu et al., 2019), cucumber (Zhao and Zhang, 2015), and barley (Gupta et al., 2021) has been reported. In our study, *T. harzianum* ST02 significantly increased sweet sorghum growth and grain yield in saline soil. These results were in agreement with our previous finding that *T. harzianum* ST02 increases the survival rate, plant height, and fresh weight the chlorophyll content and net photosynthetic rate of tomato seedlings under 200 mM NaCl stress (Zhao et al., 2021). Previous studies had found that *T. longibrachiatum* T6 increased IAA production and ACC-deaminase activity to enhance tolerance to NaCl stress and promote wheat growth (Zhang et al., 2019).

The main fermentable sugars in sweet sorghum stems are sucrose, fructose, and glucose (Almodares and Hadi, 2009); sucrose is the main sugar, accounting for 85% of the total content, and it is mainly accumulated through photosynthesis (Gowik et al., 2011; Sui et al., 2015). Under saline conditions, plants accumulate soluble sugars to promote osmotic regulation and thus improve dehydration tolerance (Farhangi-Abriz and Torabian, 2017). In our field trial, there was a significant increase in sugar content by inoculating ST02 than control (**Figure 1C**).

### 4.2 Effects of *Trichoderma* on soil properties and nutrient availability in the saline soil

Salinity stress affects the development of plant roots, thus affecting the utilization of soil nutrients and inhibiting plant growth (Zhao et al., 2020). *Trichoderma* can improve plant growth, especially when the conditions were unfavorable (Khomari and Davari, 2017), possibly by improving nutrient availability and photosynthesis efficiency (Asad, 2022). Previous studies had found that inoculated chickpea with *Trichoderma* increased nutrient availability and release of growth-promoting substances to enhance chickpea growth (Rudresh et al., 2004). In this study, we found that *Trichoderma* ST02 significantly decreased soil EC by 60.63% (**Figure 1L**, p < 0.01) to enhance soil nutrient availability and promote the growth of sweet sorghum. Additionally, compared with the control, *Trichoderma* enhanced the contents of SOM and AP by 30.6% and 44.77%, respectively, and the content of AN doubled in the rhizosphere soil, indicating that nutritional conditions of the sweet sorghum rhizosphere soil were improved. This might be attributed to that *Trichoderma* could release organic acid to solubilize nitrogen and phosphorus in soil and increase soil organic matter and soil fertility (Pang et al., 2017; Velmourougane et al., 2017; Ye et al., 2020). Similar results were also found in the AK; inoculation with *Trichoderma* could increase the AK content and restrain the Na^+^ uptake, which are beneficial for alleviating salt stress (Yu et al., 2019).

### 4.3 *Trichoderma* affected the composition of soil bacterial community

The composition and diversity of the soil microbial community in the rhizosphere are crucial for soil quality and plant health (Imane et al., 2021). In this study, compared with the control, *Trichoderma* inoculation significantly changed the structure and diversity of soil bacterial communities. It significantly reduced the bacterial diversity and richness and changed the bacterial taxonomic composition (**Figure 2**).

*Trichoderma* increased the relative abundance of Actinobacteria but decreased that of Bacteroidetes in both rhizosphere soil and bulk soil. Actinobacteria plays a vital role in the decomposition of organic matter, and they could also produce antibiotics to inhibit plant pathogens in soil (Zhang et al., 2017b), which might contribute to the growth and yield improvement of sweet sorghum. At the genus level, we found that *Trichoderma* treatment significantly increased the abundance of *Arthrobacter*, *Streptomyces*, *Nocardioides*, unclassified Frankineae, and *Bradyrhizobium* in both rhizosphere and bulk soil. Among them, *Arthrobacter* is the dominant bacteria in soil as the plant growth-promoting bacteria with the ability to degrade atrazine (Bazhanov et al., 2017). In addition, Jiang et al. (2004) had isolated *Arthrobacter* strain HS-G8 with nitrogen fixation activity from soil, which is in line with our results that *nifH* gene was higher in the soil with *Trichoderma* inoculation. *Streptomyces*, *Nocardioides*, and Frankineae were the dominant bacterial groups of *Actinobacteria*. *Streptomyces* can produce antibiotics and promote the decomposition of organic matter (Qin et al., 2011). Previous studies reported that Frankineae and *Bradyrhizobium* are linked to nitrogen fixation in plants (Sellstedt and Richau, 2013; Matos et al., 2021), which are related to the increase of *nifH* gene and available N in the soils with *Trichoderma* application in the present study. In addition, ST02 treatment decreased the abundance of *Geminicoccus* in both rhizosphere and bulk soil. Previous studies found that *Gemmatimonadaceae* (genus *Gemmatimonas*) was negatively correlated with the contents of AN and AP (Li et al., 2021). This was consistent with the result that the relative abundance of *Gemmatimonadaceae* decreased with higher AN and AP contents under *Trichoderma* ST02 treatment compared with control. These results suggested that the nitrogen cycle was enhanced in the *Trichoderma* treatment, thus promoting the uptake of nitrogen nutrients in sweet sorghum.

### 4.4 *Trichoderma* shift the abundance of N-cycling gene

*Trichoderma* can promote the nitrogen uptake and accumulation in tobacco and leafy vegetables (Singh et al., 2018; Visconti et al., 2020) and increased nitrate nitrogen in the soil (Cheng et al., 2020; Wu et al., 2022). In this study, we found that the mean abundance of *nifH* gene was greater than any other tested N cycling genes. *Trichoderma* ST02 increased the relative abundance of unclassified Frankineae and *Bradyrhizobium*, which were closely linked to nitrogen fixation and might explain the higher *nifH* gene abundance.

In addition, we also studied the effects of *Trichoderma* inoculation on the abundance of nitrification genes (bacterial *amoA* and archaea *amoA*) and denitrification genes (*nirS* and *nosZ*), which can change the contents of NH_4_^+^-N and NO_3_^−^-N in the soil (Wu et al., 2022). The first step of nitrification is ammonia oxidation, which is driven by ammonia-oxidizing archaea (AOA) and ammonia-oxidizing bacteria (AOB) (Shafiee et al., 2021). In this study, we found that the abundance of bacterial *amoA* and archaeal *amoA* genes increased significantly in the rhizosphere soil compared with control. However, in bulk soil, only bacterial *amoA* increased and the abundance of archaea *amoA* decreased significantly compared with control (**Figures 3B, C**). Previous studies have shown that the content of organic matter was the main factor affecting the growth of ammonia-oxidizing microorganisms and their ammonia-oxidizing function (Kirchman, 2012; Ouyang et al., 2018), and the lower AOA : AOB ratio in the fertilized treatments was associated with higher soil nitrification potential (Sun et al., 2015). Our results indicated that *Trichoderma* changed the abundance of *amoA* and promoted the conversion of ammonia nitrogen to nitrate nitrogen. For denitrification genes, ST02 had no significant effect on *nirS* and only increased the abundance of *nosZ* in rhizosphere soil.

## 5 Conclusion

This study demonstrated that *T. harzianum* ST02 significantly improved the growth and productivity of sweet sorghum. The promoting effect of *Trichoderma* was mainly attributed to the increase of the available nutrients and the reduction of EC in the rhizosphere soil, and the modified soil microbial community. *Trichoderma* inoculation of sweet sorghum remarkably increased the relative abundance of bacterial genera such as *Arthrobacter*, *Nocardioides*, unclassified Frankineae, and *Bradyrhizobium*, which were linked to nitrogen fixation. Therefore, the results of our study showed that *T. harzianum* ST02 had the potential to improve soil properties and enhance plant productivity in the saline soil.

## Data availability statement

The data presented in the study are deposited in the CNGB Sequence Archive, accession number CNP0004730.

## Author contributions

YLW: Conceptualization, Data curation, Formal Analysis, Funding acquisition, Investigation, Methodology, Project administration, Resources, Software, Supervision, Validation, Visualization, Writing – original draft, Writing – review & editing. HY: Conceptualization, Data curation, Methodology, Writing – review & editing. JH: Data curation, Formal Analysis, Investigation, Methodology, Project administration, Software, Validation, Writing – review & editing. HL: Data curation, Investigation, Methodology, Resources, Writing – review & editing. ZZ: Conceptualization, Data curation, Formal Analysis, Methodology, Resources, Writing – review & editing. YZW: Conceptualization, Data curation, Investigation, Project administration, Software, Writing – review & editing. JL: Conceptualization, Funding acquisition, Investigation, Methodology, Project administration, Resources, Supervision, Writing – review & editing. YZ: Conceptualization, Investigation, Methodology, Resources, Software, Supervision, Visualization, Writing – review & editing. KY: Data curation, Formal Analysis, Methodology, Writing – review & editing. HTY: Conceptualization, Formal Analysis, Funding acquisition, Project administration, Resources, Supervision, Validation, Writing – review & editing.
